# Review of "*Fish diseases*, Volumes 1 and 2." by Jorge C. Eiras, Helmut Segner, Thomas Wahli and G.B. Kapoor

**DOI:** 10.1186/1756-3305-2-46

**Published:** 2009-10-02

**Authors:** E Tellervo Valtonen

**Affiliations:** 1 Department of biologial and environmental science, University of Jyväskylä, PL 35 (Ambiotica) 40014 Jyväskylän yliopisto, Finland

## Abstract

Book review of "Fish diseases, Volumes 1 and 2." by J. C. Eiras, H. Segner, T. Wahli and G.B. Kapoor.

## Book review

Parasitic diseases and parasites in general are enormous in numbers. In fact, parasites alone encompass more than half of all known animal species. Accordingly, the agents causing fish diseases are diverse, including viruses, bacteria, fungi and parasites.

This set of two books aims to present disease-causing agents from all these major categories. The topic is wide as diseases occur in freshwater and marine aquacultures as well as in wild fish. Problems with disease are increasing due to intensified fish farming and to many changes in the environment. Moreover, problems vary considerably between different geographic regions, aquaculture systems, fish species, and societies. As such, the topic of these books is most demanding and ambitious. Clearly, nobody can single-handedly handle all aspects of the most important fish diseases even in two books.

The editors have succeeded in inviting qualified scientists to write chapters on each disease group. The editors, three from Europe and one from India, have invited 28 scientists from 12 countries to write the books' 21 chapters. Sixteen scientists are from Europe, seven from the USA, two from Australia, and one each from Canada, South Africa, and Israel. The author(s) of each chapter often come from the same country or institute, and in a few cases, this is reflected in the content of the chapter; the problems of his/her region are given considerable attention. It might have been beneficial to add one more scientist representing a different system or latitude to each topic.

Every group of disease agents has its own peculiarities, e.g. viruses and bacteria need a different kind of approach than parasites. Accordingly, there is much variation in the composition of the chapters. Nonetheless, I found the treatment of different groups disproportionate in some cases. For example, about 200 pages in three chapters are given to viral diseases (in salmonids, cyprinids and cultured marine fish, all comprehensive and good chapters), but this is not in balance with the 40 pages spent on bacterial diseases. Up-to-date information on many important bacterial diseases was missing, such as yersiniosis (caused by *Yersinia ruckeri*), columnaris disease (caused by *Flavobacteria columnaris*) and cold water disease (caused by *Flavobacteria psychrophilum*). The two latter species are causing increasing problems for the fish farming industry in wide areas of the Northern hemisphere. New molecular tools have improved diagnosis of these diseases and made it possible to conduct experiments, which I had expected to be reviewed. There has apparently been a delay between writing the articles and printing the books.

The chapter on fungal diseases is interesting, comprehensive and well written. It also includes some discussion on therapy and control methods. Parasites in volume 1 are handled in five chapters: Microsporidia, Ameboid protists, Flagellates, Apicomplexans and Ciliaphora. All of them are enjoyable to read, well written and up-to-date. Flagellates are handled especially comprehensively, as they are important disease agents in freshwater and marine aquaculture. I would have liked a similarly thorough handling of Ciliaphora. The flagellate chapter takes nearly 100 pages while the ciliate chapter is only about 30 pages long. The ciliate causing white spot disease (*Ichthyophthirius multifiliis*) is discussed at length. However, the methods described to treat it are not up-to-date. It is not mentioned, for example, that malachite green is strictly prohibited by EU legislation. In large fish farms, at least in the Northern hemisphere, parasite numbers are kept low with formalin baths for 3-4 weeks, during which time fish develop immunity. This common practice is also not discussed.

In volume 2 there are 10 chapters; seven on particular parasite groups, as well as chapters for non-infectious diseases, environmental diseases and guides for aquaculture veterinarians (why not for biologists working at farms?). The chapters in this volume are more demanding in the sense that there is much variability in the biology of the groups handled, which are Myxozoa, Monogenea, Digenea, Cestoda, Acanthocephala, Nematoda and Crustacea. These groups also vary greatly in their importance as disease agents. However, if the book had been restricted to only those species causing problems in aquaculture, it would have been much more boring. Parasites also have important effects on wild fish, though they are not as dramatic as in cultured fish. I very much enjoyed that for most of the important taxonomic groups several aspects of their biology were covered, including general morphology, life cycles, classification, host range and distribution, diseases caused in freshwater and marine systems, and in different fish groups, treatment and prophylaxis, as well as prospects for future research. These are not handled in all cases, however; especially in the chapter on Cestodes pathogenesis is emphasized over the other topics.

The fact is that fish stocks all over the world are threatened by overfishing. This means that both freshwater and marine aquaculture will expand and intensify. At the same time, global warming and other environmental changes will influence the emergence and occurrence of diseases. Fish farms are places where many epidemiological factors may drive disease agents towards higher virulence. Given these challenges, these books are helpful for fish farmers, researchers, university students and state officials working on fish diseases.

## Competing interests

The author declares that she has no competing interests.

